# Mosquirix™ RTS, S/AS01 Vaccine Development, Immunogenicity, and Efficacy

**DOI:** 10.3390/vaccines10050713

**Published:** 2022-04-30

**Authors:** Aroosa Younis Nadeem, Adeeb Shehzad, Salman Ul Islam, Ebtesam A. Al-Suhaimi, Young Sup Lee

**Affiliations:** 1 Department of Biomedical Sciences, School of Mechanical and Manufacturing Engineering (SMME), National University of Sciences and Technology (NUST), Islamabad 44000, Pakistan; ayounis.phd21smme@student.nust.edu.pk (A.Y.N.); adeeb.shehzad@smme.nust.edu.pk (A.S.); 2 Department of Pharmacy, CECOS University, Peshawar 25000, Pakistan; salmanulislam@cecos.edu.pk; 3 Biology Department, College of Science and Institute for Research and Medical Consultations (IRMC), Imam Abdulrahman Bin Faisal University, Dammam 31441, Saudi Arabia; ealsuhaimi@iau.edu.sa; 4 BK21 FOUR KNU Creative BioResearch Group, School of Life Sciences, College of Natural Sciences, Kyungpook National University, Daegu 41566, Korea

**Keywords:** parasite, infection, vaccine, malaria

## Abstract

Malaria is a parasitic infection caused by bites from *Plasmodium falciparum* (*P*. *falciparum*)-infected mosquitoes with a present scale of symptoms ranging from moderate fever to neurological disorders. *P. falciparum* is the most lethal of the five strains of malaria, and is a major case of morbidity and mortality in endemic regions. Recent advancements in malaria diagnostic tools and prevention strategies have improved conjugation antimalarial therapies using fumigation and long-lasting insecticidal sprays, thus lowering malarial infections. Declines in the total number of infected individuals have been correlated with antimalarial drugs. Despite this, malaria remains a major health threat, affecting more than 30 million men, women, and children around the globe, and 20 percent of all children around the globe have malaria parasites in their blood. To overcome this life-threatening condition, novel therapeutic strategies, including immunization, are urgently needed to tackle this infection around the world. In line with this, the development of the RTS, S vaccine was a significant step forward in the fight against malaria. RTS, S is a vaccine for *P. falciparum* in which R specifies central repeat units, T the T-cell epitopes, and S indicates surface antigen. The RTS, S/AS01 malarial vaccine was synthesized and screened in several clinical trials between 2009 and 2014, involving thousands of young children in seven African countries, showing that children who received the vaccine did not suffer from severe malaria. Mosquirix™ was approved by the World Health Organization in 2021, indicating it to be safe and advocating its integration into routine immunization programs and existing malaria control measures. This paper examines the various stages of the vaccine’s development, including the evaluation of its immunogenicity and efficacy on the basis of a total of 2.3 million administered doses through a routine immunization program. The protection and effectiveness provided by the vaccine are strong, and evidence shows that it can be effectively delivered through the routine child immunization platform. The economic cost of the vaccine remains to be considered.

## 1. Introduction

Malaria is a parasitic illness mostly found in tropical and subtropical regions, and is transmitted to humans by mosquitoes. The signs and symptoms of malaria include fever, shivers, stomach pain, nausea, muscle aches, tiredness, and lethargy. The incubation period of malarial infection is 7–14 days. *P. falciparum* remains the most lethal of all species infecting the population, and presents a huge health concern in malaria-endemic regions [1,2].

In outbreaks of *P. falciparum*, 87% of infected individuals remain asymptomatic, and are considered to be reservoirs for infection transition as a result of the shortage of efficient diagnostic routes for malaria in endemic countries [3]. People with mild infection develop disease manifestations like malaria, and the fatality rate of people with mild infection is 1%. People with a severe malarial infection suffer from problems such as significant obtundation, breathing difficulties, numerous seizures, prostration, convulsions, irregular blood discharge, and jaundice [2].

Patients suffering from acute malarial infection who do not receive proper medication and treatment have a 90% mortality rate, whereas those who receive proper treatment have a 20% mortality rate [4]. The risk of death from severe malarial infection increases with age and the severity of the disease. Investigations in Indonesians [5] and Indians [6] including adults and children below five years of age showed that early treatment can mitigate severe malarial infection [7]. Some forms of severe malarial infection have a more dire outlook than others [8]. It has been reported that the development of an effective antimalarial vaccine requires the integration of multiple areas of research, like (1) deep understanding of the biological life cycle of *P.falciparum*; (2) specification of objectives for the study of a wide scale of strains; (3) specification of an appropriate adjuvant to stimulate immunogenicity in terms of both quantity and quality against the antigen, regardless of the effect of age [9]; and (4) identification of an appropriate ‘perfect mix’ (such as the type of emulsion, nanoparticles, liposomes) as a vaccine carrier [10]. In essence, a deep investigation of the biological infestation of the parasite will reveal important data. Managing these factors will result in the provision of suitable assistance to poor responders to the vaccine [3].

## 2. World Health Organization Statistical Analysis of Malarial Infection

In the world malarial report 2018, produced by WHO, it was revealed that the numbers of people affected by malaria were plateauing in 2017; there were an estimated 219 million cases of malaria, compared to 217 million in 2016 [11]. According to the report, 50% of malaria cases of the year 2017 occurred in India, Nigeria, Uganda, Congo, and Mozambique [11]. Malaria cases rose in 2017 compared to the previous year, raising concerns about recent advances in the disease. As per the report, it was claimed that 607,000 deaths were reported in 2010 due to malarial infections, whereas 451,000 deaths were reported in 2016. The mortality rate decreased to 435,000 deaths worldwide in 2017. Reports have shown that there a gradual decrease in the mortality rate is occurring [11].

Children under the age of five continue to be the most vulnerable. Malaria is the leading cause of mortality in children, accounting for 61% of all malaria-related fatalities in 2017. Even though post-2010 mortality rates decreased, no serious steps have been taken to improve techniques and technologies with the aim of eradicating this disease [11]. Malaria prevention techniques are developed to reduce the number of people who die from the disease.

## 3. Transmission of Malarial Infection

Of the 100 species of plasmodium, five common species are responsible for causing malarial transmission in human beings, the most prevalent and fatal of which is *P. falciparum*. *P. vivax* is a major source of illness and fatality in many parts of the globe. Malarial parasites are single-celled eukaryotes that infect a variety of insect and vertebrate taxa, including mammals and birds. The life cycle of malarial parasites begins with the growth of plasmodium in an insect species that relies on a host’s body and inserts harmful parasites into the bloodstream. Diagnostic indications of malaria develop when it causes the lysis of RBCs in the host.

The circumsporozoite protein (CSP) extracted from *Plasmodium* spp. is a diagnostic antigen tool used as a biomarker to control seasonal alterations in malarial infection. By applying *P. vivax* CSP antibody using the ELISA method, epidemiological features were studied within the populations of many affected countries during the period 2017–2018. *Pv*CSP is a helpful antigen to detect the extant of spread of malarial infection. Since the antibody is relatively transient, it can act as sero-epidemiological factor for estimating the extent of malaria transmission in the present year [12].

Female anopheline mosquitoes transmit *P*. *falciparum* sporozoite-stage parasites to the host. Venous circulation of blood helps them reach the host’s liver, which is where offspring are produced [13]. The number of progenies they produce is between 30 and 40,000, and takes about 6 days. Infected liver cells then rupture, and merozoites are discharged following the pre-erythrocytic stage, which shows no clinical symptoms. They infect erythrocytes and generate 16–24 merozoites during a 2–3-day propagation period [14,15].

Pyrexia, nausea, tiredness, and lethargy are signs of the rise in cytokine levels due to the bursting of infected red blood cells (RBCs), which leads to severe malarial infection causing renal disease, jaundice, hypoglycemia, coma, and death [16,17].

**Figure 1:**
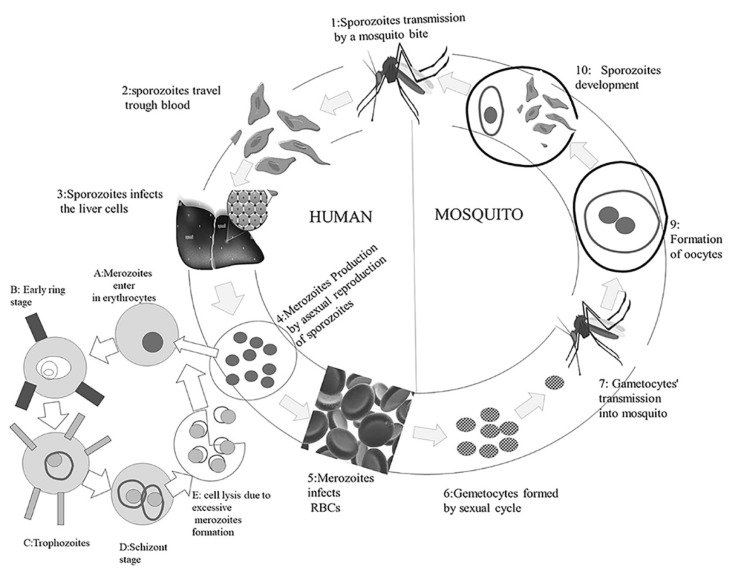
Transmission of malarial infection from mosquito to human.

The initial phases of malarial illness are characterized by recurrent infections and pyrexia, with a periodicity that is unique to different malarial species. The newly produced merozoites are discharged in the bloodstream throughout these periods. In addition to this, RBC infection can be of two different types: it can be an erythrocytic invasion by asexual merozoites, or an infections by male or female gametocytes that can be consumed by a mosquito during blood-feeding, which then mix in the insect’s midgut, generating diploid recombinant progeny [18,19]. Figure 1 explains this transmission of malarial infection from mosquito to human.

## 4. Methods to Prevent and Control Malarial Infections and Their Limitations

The transmission of malarial infection is a major cause of illness and fatality, and a variety of different methods have been used for its prevention and control. Until 1600, the exact cause of malaria was not known, and “medicine men” suggested quinine as the only treatment for malaria [20]. It was discovered in 1897 that malaria was a vector-borne disease caused by Anopheles mosquitoes. Afterwards, people adopted preventive measures such as placing mosquito nets around beds and the use of insecticidal sprays. Although these preventive measures were cost effective, they were not enough to control malaria. Subsequently, various new drug molecules were discovered to combat malaria [21,22,23]. The emergence of resistance against antimalarial drugs is calling into question the efficacy of the available drugs. To cater to all of these problems, the development of a potential vaccine against malaria is essential, and is expected to combat malaria globally [11]. The most effective way of stopping the transmission of malarial infection is a vaccine that works with optimal efficiency and efficacy, working in a way that starts by inhibiting the initial phase of pathogen growth followed by the prevention of the subsequent stages.

## 5. Vaccines—The Most Efficient Tool for Combating Malarial Infection

A successful malaria vaccine would be the most significant weapon in countering the disease’s massive impact on socio-economic domains. Vaccines are one of the most effective public health interventions, since they benefit everyone [24,25]. Compared to other human initiatives, such as cleanliness and sanitation, making people immune against infectious illnesses is one of the key components of global health measures [26]. A new phase IIb clinical trial registered as NCT03896724 reports that the antigen R-21, a malarial vaccine candidate created by the University of Oxford using Novavax Inc.’s Matrix-M adjuvant, demonstrated 77% efficacy in children [27].

The United Nations’ (UN) sustainable development goal #3 aims to reduce deaths and infections caused by malaria in acknowledgment of the worldwide relevance of malarial infection [28]. Another of the UN’s sustainable development goals is to enhance quality of life by reducing malaria’s mortality by 90% by the year 2030 [28]. The eradication of malarial infection in not less than 35 affected countries is another part of this aim [11]. Bed nets to avoid mosquitoes and other insects, larval source monitoring, and long-lasting insecticidal sprays are a few of the advancements in malarial infection control management.

Antimalarials are given to susceptible people on an intermittent basis to remove malaria-causing parasites and avoid illness. Certain other projects in the works involve mass medication distribution and bioengineered mosquitoes. The most significant task that has been performed in this regard is the research carried out on the execution of the first malarial vaccine directed by the WHO [26,29,30]. This research aimed to determine possible ways of optimizing the impact of the RTS, S vaccine for the health of the general population. The RTS, S vaccine is the most sophisticated malarial vaccine so far [29].

People, including those who have been immunized with shots of the RTS, S/AS01 vaccine, are vulnerable to repeated attacks of malarial infection due to site-specific genetic diversity. Although there is no documented immune responsiveness that linking *P*. *falciparum* to different species of malaria, this field of study requires further intensive research. Gene diversity is the main reason for the evasion and resistance of vaccines against diverse strains, and this was recently indicated in a study in which reduced effectiveness was demonstrated in those variants possessing heterologous sequences when compared with the pattern of the RTS, S vaccine [30]. The categorization of vaccines for malaria is based on different phases of parasite development. The most effective vaccine is a pre-erythrocytic vaccine, such as RTS, S, which directly arrests the circumsporozoite proteins present on the surface of sporozoites. The RTS, S vaccine arrests the *P*. *falciparum* parasites and destroys them before they enter the liver, where they could develop.

The first and foremost vaccine for malarial infection, which was evaluated up to phase III clinical trials to check its efficacy and efficiency, was RTS, S/AS01. The RTS, S/AS01 vaccine was also among the first to be evaluated in endemic zones in regular vaccination drives. A study, conducted upon 8922 patients with malaria, showed that administration of vaccines to children between the ages of 5 and 7 years produced a rate of 36% effective immunization against malaria. All of these children received four shots of the RTS, S/AS01 vaccine at months 0, 1, 2, and the booster shot was administrated at the age of 20 months. They were followed up for a period of four years. The registered number for this clinical trial is NCT00866619.

Clinical trials upon newborns between the ages of 6 and 14 weeks showed that the vaccine’s effectiveness was even lower than the efficiency attained in children of 5–7 years. Low efficiency of RTS, S/AS01 vaccine in infants was reportedly due to the immature immune system and the intervention of maternal antibodies in neonates [31].

In places with high endemic rates, RTS, S/AS01 was the most promising vaccine. The vaccine helps to drastically decrease the incidence of malaria, hospitalization rates, and other requirements in the treatment of infection.

The RTS, S/AS01 vaccine was acknowledged by the WHO, and received a grant for further assessment and appraisal to ensure its effectiveness before including it in mass immunization. An immunization drive to evaluate the effectiveness of the vaccine was undertaken in different endemic regions within Malawi, Ghana, and Kenya [32,33].

## 6. Development of RTS, S/AS01 Vaccine

The Walter Reed Army Institute of Research (WRAIR) and GlaxoSmithKline (GSK), in 1987, developed the RTS, S malarial vaccine. The National Institute of Health and WRAIR had found that sporozoites that had been exposed to radiation elicited an immune response, hence producing a response for which the target was the CSP (circumsporozoite) antigen. Furthermore, they had determined the genetic pattern of the CSP antigen, sequenced it, and produced the genetic clone [34,35,36,37].

Following the production of Energix- B™, a vaccine used to immunize against the Hepatitis B virus, a research team from GSK created the RTS, S vaccine for malarial infection. For the NF54 strain of *P*. *falciparum,* the researchers utilized the surface antigen of Hepatitis B as a CSP carrier matrix and inserted CSP’s C terminals, which carry epitopes for both B and T cells [38]. The “R” in RTS, S vaccine stands for the central repeat region, made up of one chain of amino acids N-acylneuraminate-9-phosphatase (NANP) amino acid tandem repeat tetrapeptides, ‘T’ stands for the immunodominant segregated epitopes of T lymphocytes, and ‘S’ stands for the surface antigen of Hepatitis B [39,40,41].

Coexpression of RT peptides in *Saccharomyces cerevisiae*, along with the fusion of the N terminus surface antigen of Hepatitis B, resulted in the production of entities analogous to the virus, possessing surface CSP and S. The additional “S” component is the Hepatitis B surface antigen, which is combined with the RTS component. The RTS, S antigen has a highly recurrent structure, which is why immunization is believed to aid in the defense mechanism by producing anti-CSP antibodies and T cells [42].

The RTS, S vaccine was exposed to a wide range of adjuvants by GSK researchers, almost all of which were found to be effective. ASO4 is the combination of an adjuvant—alum versus alum + 3-O-desacyl-4′- monophosphoryl lipid A (MPL)—and RTS, S. The first clinical trial of the RTS, S vaccine was conducted using this combination. It was found that, as compared to CHMI, AS04 showed enhanced efficacy and safety [43].

On the basis of studies performed in rhesus monkeys, it was found that six competing adjuvants tested with the RTS, S vaccine abolished ASO2A, which is a combination of oil-in-water emulsion, monophosphoryl lipid A, and the extract from Quillaja saponaria. The RTS, S vaccine provided extraordinary immunization and CMI reactivity [42]. When CHMI was studied with the RTS, S vaccine in clinical research, all adjuvants, such as oil-in-water emulsifications (AS02, AS03) and AS04, were evaluated to determine their activity. The concluding results showed that AS02A offered a better immune response compared to CHMI, while both oil-in-water emulsifications, that is, AS03 and AS04, showed a similar immune response [44]. These results were followed by attaining a stable ASO2A, which was achieved by creating a lyophilized RTS, S formulation that demonstrated similar effectiveness toward CHMI [45,46].

Following that, further improvements in the immune response of the RTS, S vaccine were made using a novel adjuvant approach called AS01, which is the combination of QS-21, liposomes, and MPL. The clinical study of young people in Kenya validated that ASO1 had a better immune response than ASO2. ASO1 produces enhanced response in anti-CSP antibody and CD4+ T cells [47]. These validated results allowed researchers to evaluate the RTS, S/AS01 vaccine in phase 2 trials, which further led to its evaluation in phase III clinical trials [48,49,50,51,52].

## 7. Phase III Clinical Trial of RTS, S/AS01 Vaccine

Between 2009 and 2014, the malarial vaccine RTS, S/AS01 was tested in seven African regions, involving 15,459 individuals, under the registration number NCT00866619 [53]. Among them, 8922 were children aged 5–17 months and 6537 newborns aged 6–12 weeks. At the commencement of the vaccination, subjects underwent random immunization to receive three shots of vaccine at months 0, 1, and 2, a booster shot at the 20th month, or a comparator vaccine [54].

The trial was split into two parts: a double-blind phase that lasted from 0 to 32 months, and an extension phase that lasted from 33 months to the end of the trial. The average estimated observational period for children aged 5–17 months was 48 months, whereas newborns aged 6–12 weeks had an observation period of 38 months. The immunization shots were administered intramuscularly. As part of the long-term vaccination program, infant immunizations were paired with administration of oral polio and parenteral DTP-containing vaccines.

This Phase III vaccine study aimed to evaluate RTS, S/AS01 effectiveness in two age groups, the effectiveness of a booster dose, and to analyze its long-term efficiency after the termination of the extension phase. Table 1 sums up the information about the efficiency of phase III clinical testing of RTS, S vaccine.

The main outcome was the identification of clinical symptoms of malaria by passive approaches and *P. falciparum* asexual parasitemia strain, including active infection markers or indicators with no treated diseases over the time duration of one year. A sickness with an elevated fever of at least 37.5 °C and the existence of *P. falciparum* asexual parasitemia was classified as severe malarial infection [54,55].

In an analysis after subjecting patients to the third dose of the vaccine, it was found that the coprimary endpoints of efficacy to clinical malaria were 55.8% (97.5% CI 50.6–60.4) in children aged 5–17 months and 31.3% (23.6–38.3) in infants aged 6–12 weeks. This analysis took a total time of four years from March 2009 to January 2011. These analytical data were obtained using the passive case finding method [55].

The protocol was later amended to extend the follow-up period to 31 December 2013 (median follow-up 48 months for children and 38 months for young infants). Four shots of RTS, S/AS01 vaccine showed 25.9% effectiveness against malarial infection in newborns aged 6–12 weeks and 36.3% in children aged 5–17 months. The vaccine’s effectiveness for malarial infection declined with time for both categories and programs, although the drop-off in efficiency with four dosages is comparatively slow. The effectiveness of the vaccine was reduced in 6–12-week-old newborns compared to in 5–17-month-old children. Children between the ages of 5 to 17 months were prone to develop a febrile seizure seven days, when they were administrated with their third shot of vaccine. This was a temporary impact, and all the afflicted children healed within seven days. It was suggested that the fourth shot, which was a booster shot, enhanced the vaccine’s efficacy. The efficacy of the vaccine was determined using negative binominal regression in the case of clinical malaria [55].

## 8. Production of RTS, S/AS01 Vaccine

In 2001, GSK and the Program for Appropriate Technology in Health collaborated to produce the malarial vaccine RTS, S with the goal of combating malaria in Sub-Saharan Africa. Industrial manufacturing of pure mass antigen RTS, S consists of a few stages, including feed-batch fermentation of the modified yeast variant *Saccharomyces cerevisiae*, RIX4397, which is collected from a two-tier cell bank by a variety of different processing steps such as cell collection, disruption, separation, and filtration.

Various kinds of chromatography apparatuses, ultracentrifuges, and filters are used in the purification process. The maintenance and storage temperature of RTS, S is −70 °C. In the formation process, standard control conditions are implemented to ensure product preservation, output, and quality during the production process. For a minimum of three consecutive sets, the procedure must be consistent to ensure the validity of the factor used during manufacturing of the vaccine [56].

The production of GSK biologicals’ AS01 conformation involves the following steps: a combination of liposome with PO4/NaCl buffer solution is incorporated into QS-21 liquid substance; then, the pH of the formulation is assessed; and after sterile filtering, it is poured into the containers.

The rebuilt RTS, S/AS01 adjuvant framework is an opalescent, translucent-to-pastel brown injectable solution devoid of contaminants. RTS, S is available in pellet or powder form, preserved in a glass tube of nearly 3 mL stopped with a rubber cork and capped with an aluminum lid. The other is two dosages containing the AS01 liquid formulation, stored in a glass tube of 3 mL, stopped with a rubber cork, and capped with an aluminum lid. The two shots are injected intramuscularly, one from each glass tube [56].

The genetic variation in the two protein components of yeast cells, i.e., RTS and S, and their autonomous merging produces entities that resemble the virus with CSP and S patterns [34]. For further exploration and phase IV evaluation, a considerable quantity of vaccines is needed. GSK is manufacturing and donating the vaccine for implementation in current trials. Although the vaccine was not designed for sale within the EU, it meets the same quality requirements as goods sold within the EU. In July 2015, the CHMP completed a research analysis of the malarial vaccine and provided a favorable judgment that the overall analysis was sound.

The advantages may be highly relevant for youngsters in endemic malarial regions, according to the CHMP. The WHO Strategic Advisory Group of Experts on Immunization (SAGE) and the Malaria Policy Advisory Group (MPAC) evaluated Phase III clinical results for the RTS, S malarial vaccine in October 2015. The WHO has suggested preliminary investigations with the RTS, S/AS01 vaccine utilizing a four-dosage schedule in three to five malaria-endemic areas of Sub-Saharan Africa with moderate to high rates of malarial infection.

The vaccine is presently being tested in different malaria-endemic regions in children between 5 and 17 months of age under the clinical trial number NCT00866619. The findings of these studies should help decision makers, regulators, and sovereign states make decisions regarding the licensing of RTS, S/AS01. Children who were administrated only three shots of RTS, S/AS01 vaccine are prone to higher malarial risk in later stages. This was validated by research conducted for seven years, which concluded that vaccine effectiveness decreased with time in newborns, with an age limit between 5 and 17 months [57].

## 9. Economic and Community Health Issues

Gavi’s objective was to save lives and safeguard public health by promoting equal vaccination among underdeveloped countries [58]. The RTS, S/AS01 vaccine falls in line with that mission. PATH and GSK consider and accept that the availability of vaccination to children should be ensured at zero cost. Current systems that provide free vaccinations to children in Africa could potentially aid in achieving this aim. GSK has said that the price will be based on production costs with a 5% return to be utilized in funds used for vaccines for other infections [58].

Although the RTS, S/AS01 vaccine has low efficacy, it nevertheless has considerable advantages regarding people’s general health. Among every 1000 children who were given four vaccination shots, a total of 1744 cases of malarial infection were avoided. Furthermore, according to data from phase III clinical trials conducted under the direction of the WHO, it was found that four doses of the vaccine would avoid 116,480 instances of malarial infection and 484 fatalities per 100,000 immunized children.

In the malaria-endemic regions where the infection rate is 10%, RTS, S/AS01 immunization is considered one of the most practical measures, compared to other measures to save children from getting malarial infection such as nets treated with insecticide and temporary malaria chemoprophylaxis [58].

The majority of the participants in the study were willing to pay for malaria vaccinations for children at significantly less than the profile cost. Therefore, it helps policy makers to be aware of the price issue before specifying the price of the vaccine [59]

## 10. Vaccine Administration via Intramuscular Method

RTS, S/AS01 is administered via intramuscular injection as a lyophilized suspension. Children are currently prescribed four shots of vaccine. According to the administration schedule of the vaccine, the first shot should be given to the infant at the age of five months, and the rest should be given with one-month gaps until the age of 9 months. After that, children aged 15–18 months should be given a fourth shot of vaccination.

## 11. Immuno-Stimulation

The immunogenicity of RTS, S has been intensively studied, and successful attempts were made to link immunological behavior to the vaccination in order to safeguard public health [60,61,62,63,64]. Anti-CSP antibody levels in opposition to the repeat sequences of NANP and higher levels of antibodies and responsiveness have been linked to a lower rate of malarial infection, although no protective cutoff value has been determined [65]. An increase in the levels of CD4+ T cells, which belong to the immune system has also linked RTS, S/AS01 inoculation with the prevention of disease [66].

Immunological studies in susceptible groups such as HIV-positive children have also been performed to evaluate the responses. Clinical investigations have been performed on children living in Kenya suffering from first- or second-stage HIV, declared by the WHO to have higher antibody (anti-CSP) counts when compared with control groups. Compared to infants, older children showed higher immune response generation via vaccine induction in Phase III testing. Anti-CSP antibody levels increased post receipt of the booster shot of the vaccine. The study showed that antibody count decreased from 318.2 EU/mL to 34.2 EU/mL in children between the ages of 5 and 17 months who were not administered the booster shot [55]. The antibody count fell further to 52.4 EU/mL and 19.3 EU/mL, respectively, after one year.

## 12. Cross Defensive Behavior of Vaccine

These CSPs efficacy in providing protection against some malarial strains is similar to the RTS, S, and *P. falciparum* vaccines. This is further validated by a study showing that variations in the sequence of CSPs decreases the effectiveness of vaccines [30].

## 13. Public Health Awareness Campaigns about the Side Effects and Advantages of Vaccines

Malarial immunizations with an adjuvanticity profile comparable to that of regular vaccinations are more appropriate to be chosen by parents of children residing in areas with higher rates of malarial infection. The advantages and disadvantages require detailed descriptions, as well as community education drives to enlighten people about the dangers and advantages of immunization. All other preventive measures, such as bed nets, health care, and encouragement of treatment-seeking behavior, have been shown to lessen the likelihood of malarial infection and fatality, but they cannot be used in lieu of vaccination. Critical appraisal of behavioral interventions following immunization is part of the ongoing RTS, S/AS01 execution research efforts.

## 14. Recent Inventions in Malarial Vaccination

The first malarial vaccine to have received positive approval from the European Medicines Agency’s CHMP is RTS, S/AS01. It specifically targets the malarial strain *P. falciparum*, and has progressed to Phase III clinical testing.

PfSPZ Vaccine and R21 are two more potential immunizations against the *P. falciparum* strain of malaria. To determine their effectiveness, these medications are still being evaluated in people suffering from malaria in comparison with those not suffering from malaria [26,67]. However, all of these malarial vaccines were evaluated and mentioned in WHO Rainbow Table 48.

The PfSPZ Vaccine works against the *P. falciparum* NF54 strain. It is a complete sporozoite vaccine that is active and enervated by rays [68,69,70]. The time-based evaluation revealed 52% effectiveness toward severe, diverse *P. falciparum* strain in people of Africa who were suffering from malaria when they were administered five shots of 2.7 × 105 vaccines in 6 months [71].

The vaccine aims to deter malarial illness by inhibiting the pre-erythrocytic sporozoite stage of *P. falciparum* is PfSPZ. Bioko Island in Equatorial Guinea conducted major phase III clinical investigation studies for the further analysis of the vaccine. It protects against the infection of hepatocytes in the liver by producing immune response in CD8+ T cells [72].

In comparison with RTS, S/AS01, the R21 vaccination elicits a higher anti-CSP immune reaction and a lower anti-HBsAg antibody activity. In addition, the R21 vaccine is made up of a protein with a single CSP-antigen against Hepatitis B on the protein surface, resulting in a considerably greater percentage of CSP antigen.

R21 has higher effectiveness than RTS, S/AS01, and PfSPZ because of a higher concentration of CSP for each HBsAg antibody, which may lead to improved effectiveness and defense in opposition to the CHMI parasite [64]. Preliminary data from the research showed that Montanide adjuvant processing with adjuvant Matrix-M improves safety stimulation and enhances immune cell response. R21 is still in the initial phases of development, and thus there are doubts regarding its performance against CHMI antigen [73].

Table 2 presents different types of antimalarial vaccines, along with their modes of action, differences, advantages, and disadvantages.

## 15. Future Perspectives

The introduction of the RTS, S/AS01 vaccine to combat malaria in areas where the rate of malarial infection is high is a quite hopeful prospect. The WHO has started critical research to see if giving 4.486 M. B. LAURENS dosages of vaccination to children is feasible.

When people who had not encountered malaria were inoculated with the one-fifth portion of RTS, S/AS01 after the delayed third shot, they showed enhanced defense against the CHMI antigen. On the basis of this study, children were inoculated with alternate shots of RTS, S/AS01 [74]. The foundation of the enhanced immunity depends on increased CSP-specific antibody and somatic hypermutation in immune response cells, i.e., CSP-specific B-cells [74].

Another way of counteracting the fast degradation of antibodies induced by the vaccine in susceptible populations is periodic booster shots of the RTS, S vaccine. Immunity can also be improved by using complementary adjuvants for RTS, S, because of the strong reliance of the antigen on the adjuvant. Further improvements are also possible based on the consideration of possible side effects; for example, it would be advantageous to have an adjuvant that did not raise the probability of the threat of febrile seizures in children.

GSK, a pharmaceutical firm, is still producing a vaccine for the present deployment trials. Therefore, the availability of vaccines in the long term is still unclear. Additional vaccine manufacturing and delivery delays are possible, since vaccine producers must prepare ahead of time to fulfill demand, and as the WHO has now approved the RTS, S vaccine for use, now is the time for estimating what kind of quantities will be required. Shipping and stockpiling of vaccines will also require consideration at the global, provincial, and state levels. Awareness and collaborations will undoubtedly help to solve these constraints.

The most suitable approach, which is an interesting substitute for conventional vaccination techniques, is represented by current developments in mRNA technology, enabling stable and tailored antigen production. The mRNA technology-based manufacturing of COVID vaccines opened paths toward the development of messenger RNA vaccines against malaria, which could enhance the immune response through the formation of malarial pathogenic proteins [75,76]. This specific type of vaccine enables cells to produce circumsporozoite proteins, which subsequently activate a protective immunity against malaria, using mRNA along with lipid nanoparticles, where nanoparticles are used as a molecular carrier due to their favorable clinical results in terms of safety and efficacy when co-administered with mRNA. Successful administration of mRNA-based vaccines in mice have provided substantial immunity levels, making them an intriguing option for further research. The evaluation of this technique gives us insight that a pre-erythrocytic mRNA malaria vaccine provides immunity, with a variety of parameters influencing protective effectiveness [77,78,79].

## 16. Conclusions

The first malarial vaccine approved by the WHO after evaluation of phase III clinical testing is the RTS, S/AS01 vaccine. This represents a significant landmark, after decades of testing, evaluation, research, and clinical trials. When the WHO, Gavi, and other organizations examine the vulnerability, expenditure, and operational issues while addressing the execution drive of vaccines, they should also address the possible effects on people residing in malaria-endemic zones with health issues, low income, and socio-economic barriers. In addition, future advancements, such as improved defense utilizing a staggered, segmented dosage regimen, and other alternative adjuvants, must be developed with the aim of attaining the final goal.

## Figures and Tables

**Table 1:** Percentage efficiency of phase III clinical testing of RTS, S vaccine.

Sr. No	Age of the Patient	Number of Patients	Malarial Type	Number of Dosages	Efficiency %Age
1	6–12 Weeks Aged Infants	6537	Clinical Malaria	3 Doses	18.3
				4 Doses	25.9
			Severe Malaria	3 Doses	10.3
				4 Doses	17.3
2	5–7 Months Aged Children	8922	Clinical Malaria	3 Doses	28.3
				4 Doses	36.3
			Severe Malaria	3 Doses	1.1
				4 Doses	32.2

**Table 2:** Different kinds of malarial vaccines, along with their modes of action, potentials, and limitations.

Malarial Vaccine Types	Mode of Action	Differences	Advantages	Disadvantages
**RTS, S**	It is a subunit vaccine that involves the activation of immune response by targeting the sporozoite in pre-erythrocyte stage to prevent the infection.	Its immunogen type is different from PfSPZ vaccine. It generates antigen-specific immunoglobulins and CD4+ T cells in response to the plasmodium subunit antigen. It has the potential to trigger systemic responses such as discomfort, chills, and convulsions.	The RTS, S/AS01 vaccination elicits cell-mediated and antibody-mediated immune responses that provide protection against new P. falciparum infection and blocks sporozoite colonization of hepatocytes. It prevents malarial infection as well as Hepatitis B.	It provides insufficient protection that deteriorates with the passage of time. It has prompted serious safety risks in children. Using more effective adjuvants in vaccine is recommended to establish stronger immunogenicity that persists for a longer period of time.
**PfSPZ**	It is a whole-organism vaccination that attacks sporozoites that are in pre-erythrocyte stage; it generates CD8 T lymphocytes and leads to abortion of hepatocyte infection, as well as protecting the cell.	This kind of immunogen is a radiation-attenuated whole organism. Several antigenic plasmodium strains elicit a defensive immunoglobulin, as well as CD4 and CD8 T cell responses. There are no systemic responses because of it.	It is a whole-organism vaccination, the immune response to plasmodium antigens lasts for a long time without addition of any adjuvants.	In malaria-naïve individuals, it provides only partial protection against heterologous CHMI with 7G8 strain parasites. Vaccine delivery and storage under liquid nitrogen, as well as intravenous injection, are major challenges.
**R21**	It is a type of vaccine which includes a subunit of the pathogen that targets sporozoites in the pre-erythrocyte stage, which triggers an antibody and humoral immune response.	Plasmodium subunit antigen is a form of immunogen that induces immunoglobulin and CD4 T immunological responses.	R21 is a better variant of the RTS, S/AS01 that is a subunit antimalarial vaccine and is considered the next generation antimalarial vaccine to prevent malaria. The formulation of R21 results in increased humoral immune responses to CSP due to the induction of increased B cell response.	It is still in early clinical studies; therefore, there are numerous questions about its efficacy in both malaria-naïve and malaria-experienced people.

## References

[1] World Health Organization (2018). Towards a Global Action Plan for Healthy Lives and Well-Being for All: Uniting to Accelerate Progress towards the Health-Related SDGs.

[2] Ayanful-Torgby R., Quashie N.B., Boampong J.N., Williamson K.C., Amoah L.E. (2018). Seasonal variations in Plasmodium falciparum parasite prevalence assessed by varying diagnostic tests in asymptomatic children in southern Ghana. PLoS ONE.

[3] Bonam S.R., Rénia L., Tadepalli G., Bayry J., Kumar H.M.S. (2021). Plasmodium falciparum Malaria Vaccines and Vaccine Adjuvants. Vaccines.

[4] Dondorp A.M., Lee S.J., Faiz M.A., Mishra S., Price R., Tjitra E., Than M., Htut Y., Mohanty S., Yunus E.B. (2008). The relationship between age and the manifestations of and mortality associated with severe malaria. Clin. Infect. Dis..

[5] Maitland K. (2015). Management of severe paediatric malaria in resource-limited settings. BMC Med..

[6] Aqeel S., Naheda A., Raza A., Khan W. (2020). A micro-epidemiological report on the unstable transmission of malaria in Aligarh, India. Parasite Epidemiol. Control..

[7] Ross A., Maire N., Molineaux L., Smith T. (2006). An epidemiologic model of severe morbidity and mortality caused by Plasmodium falciparum. Am. J. Trop. Med. Hyg..

[8] (2014). Severe malaria. Severe malaria. Trop. Med. Int. Health.

[9] Coffman R.L., Sher A., Seder R.A. (2010). Vaccine adjuvants: Putting innate immunity to work. Immunity.

[10] Reed S.G., Orr M.T., Fox C.B. (2013). Key roles of adjuvants in modern vaccines. Nat. Med..

[11] World Health Organization (2018). World Malaria Report 2018.

[12] Lee J., Jin K., Ahn S.K., Lee S.K., Kwon H.W., Na B.K., Kim T.S. (2021). Seroprevalence of Plasmodium vivax Circumsporozoite Protein Antibody in High-Risk Malaria Areas in Korea. Korean J. Parasitol..

[13] Siciliano G., Alano P. (2015). Enlightening the malaria parasite life cycle: Bioluminescent Plasmodium in fundamental and applied research. Front. Microbiol..

[14] Flemming A. (2015). Hitting all stages of the parasite life cycle. Nat. Rev. Microbiol..

[15] Soulard V., Soulard V., Bosson-Vanga H., Lorthiois A., Roucher C., Franetich J.F., Zanghi G., Bordessoulles M., Tefit M., Thellier M. (2015). Plasmodium falciparum full life cycle and Plasmodium ovale liver stages in humanized mice. Nat. Commun..

[16] Ashley E.A., Dhorda M., Fairhurst R.M., Amaratunga C., Lim P., Suon S., Sreng S., Anderson J.M., Mao S., Sam B. (2014). Spread of artemisinin resistance in Plasmodium falciparum malaria. N. Engl. J. Med..

[17] Sypniewska P., Duda J.F., Locatelli I., Althaus C.R., Althaus F., Genton B. (2017). Clinical and laboratory predictors of death in African children with features of severe malaria: A systematic review and meta-analysis. BMC Med..

[18] Luzolo A.L., Ngoyi D.M. (2019). Cerebral malaria. Brain Res. Bull..

[19] Su X.-z., Zhang C., Joy D.A. (2020). Host-Malaria Parasite Interactions, and Impacts on Mutual Evolution. Front. Cell. Infect. Microbiol..

[20] Achan J., Talisuna A.O., Erhart A., Yeka A., Tibenderana J.K., Baliraine F.N., Rosenthal P.J., D’Alessandro U. (2011). Quinine, an old anti-malarial drug in a modern world: Role in the treatment of malaria. Malar. J..

[21] Hill A.V. (2011). Vaccines against malaria. Philos. Trans. R. Soc. B Biol. Sci..

[22] Islam S.U., Islam S.U., Shehzad A., Sonn J.K., Lee Y.S. (2017). PRPF overexpression induces drug resistance through actin cytoskeleton rearrangement and epithelial-mesenchymal transition. Oncotarget.

[23] Shehzad A., Ravinayagam V., AlRumaih H., Aljafary M., Almohazey D., Almofty S., Al-Rashid N.A., Al-Suhaimi E.A. (2019). Application of Three-dimensional (3D) Tumor Cell Culture Systems and Mechanism of Drug Resistance. Curr. Pharm. Des..

[24] Miura K. (2016). Progress and prospects for blood-stage malaria vaccines. Expert Rev. Vaccines.

[25] Patarroyo M.E., Alba M.P., Rojas-Luna R., Bermudez A., Aza-Conde J. (2017). Functionally relevant proteins in Plasmodium falciparum host cell invasion. Immunotherapy.

[26] Draper S.J., Sack B.K., King C.R., Nielsen C.M., Rayner J.C., Higgins M.K., Long C.A., Seder R.A. (2018). Malaria Vaccines: Recent Advances and New Horizons. Cell Host Microbe.

[27] Datoo M.S., Natama M.H., Somé A., Traoré O., Rouamba T., Bellamy D., Yameogo P., Valia D., Tegneri M., Ouedraogo F. (2021). Efficacy of a low-dose candidate malaria vaccine, R21 in adjuvant Matrix-M, with seasonal administration to children in Burkina Faso: A randomised controlled trial. Lancet.

[28] World Health Organization, Regional Office for Europe (2017). Malaria: Fact Sheet on Sustainable Development Goals (SDGs): Health Targets.

[29] Targett G.A.T., Moorthy V.S., Brown G.V. (2013). Malaria vaccine research and development: The role of the WHO MALVAC committee. Malar. J..

[30] Wahid F., Khan T., Shehzad A., Ui-Islam M., Kim Y.Y. (2014). Interaction of nanomaterials with cells and their medical applications. J. Nanosci. Nanotechnol..

[31] Neafsey D.E., Juraska M., Bedford T., Benkeser D., Valim C., Griggs A., Lievens M., Abdulla S., Adjei S., Agbenyega T. (2015). Genetic Diversity and Protective Efficacy of the RTS, S/AS01 Malaria Vaccine. N. Engl. J. Med..

[32] Daily J.P. (2012). Malaria vaccine trials--beyond efficacy end points. N. Engl. J. Med..

[33] Ndeketa L., Mategula D., Terlouw D.J., Bar-Zeev N., Sauboin C.J., Biernaux S. (2020). Cost-effectiveness and public health impact of RTS, S/AS01 (E) malaria vaccine in Malawi, using a Markov static model. Wellcome Open Res..

[34] Han L., Hudgens M.G., Emch M.E., Juliano J.J., Keeler C., Martinson F., Kamthunzi P., Tegha G., Lievens M., Hoffman I.F. (2017). RTS, S/AS01 Malaria Vaccine Efficacy is Not Modified by Seasonal Precipitation: Results from a Phase 3 Randomized Controlled Trial in Malawi. Sci. Rep..

[35] Cohen J., Nussenzweig V., Nussenzweig R., Vekemans J., Leach A. (2010). From the circumsporozoite protein to the RTS, S/AS candidate vaccine. Hum. Vaccines.

[36] Kaslow D.C., Biernaux S. (2015). RTS, S: Toward a first landmark on the Malaria Vaccine Technology Roadmap. Vaccine.

[37] Shehzad A., Lee J., Lee Y.S. (2015). Autocrine prostaglandin E₂ signaling promotes promonocytic leukemia cell survival via COX-2 expression and MAPK pathway. BMB Rep..

[38] Shehzad A., Parveen S., Qureshi M., Subhan F., Lee Y.S. (2018). Decursin and decursinol angelate: Molecular mechanism and therapeutic potential in inflammatory diseases. Inflamm. Res..

[39] Leroux-Roels G., Leroux-Roels I., Clement F., Ofori-Anyinam O., Lievens M., Jongert E., Moris P., Ballou W.R., Cohen J. (2014). Evaluation of the immune response to RTS, S/AS01 and RTS, S/AS02 adjuvanted vaccines: Randomized, double-blind study in malaria-naive adults. Hum. Vaccines Immunother..

[40] Bojang K.A. (2006). RTS, S/AS02A for malaria. Expert Rev. Vaccines.

[41] Didierlaurent A.M., Collignon C., Bourguignon P., Wouters S., Fierens K., Fochesato M., Dendouga N., Langlet C., Malissen B., Lambrecht B.N. (2014). Enhancement of adaptive immunity by the human vaccine adjuvant AS01 depends on activated dendritic cells. J. Immunol..

[42] Shehzad A., Qureshi M., Anwar M.N., Lee Y.S. (2017). Multifunctional Curcumin Mediate Multitherapeutic Effects. J. Food Sci..

[43] Gandhi K., Thera M.A., Coulibaly D., Traoré K., Guindo A.B., Ouattara A., Takala-Harrison S., Berry A.A., Doumbo O.K., Plowe C.V. (2014). Variation in the circumsporozoite protein of Plasmodium falciparum: Vaccine development implications. PLoS ONE.

[44] Vreden S.G., Verhave J.P., Oettinger T., Sauerwein R.W., Meuwissen J.H. (1991). Phase I clinical trial of a recombinant malaria vaccine consisting of the circumsporozoite repeat region of Plasmodium falciparum coupled to hepatitis B surface antigen. Am. J. Trop. Med. Hyg..

[45] Garçon N., Vaughn D.W., Didierlaurent A.M. (2012). Development and evaluation of AS03, an Adjuvant System containing α-tocopherol and squalene in an oil-in-water emulsion. Expert Rev. Vaccines.

[46] Kester K.E., McKinney D.A., Tornieporth N., Ockenhouse C.F., Heppner D.G., Hall T., Wellde B.T., White K., Sun P., Schwenk R. (2007). A phase I/IIa safety, immunogenicity, and efficacy bridging randomized study of a two-dose regimen of liquid and lyophilized formulations of the candidate malaria vaccine RTS, S/AS02A in malaria-naïve adults. Vaccine.

[47] Wang Z.-B., Xu J. (2020). Better Adjuvants for Better Vaccines: Progress in Adjuvant Delivery Systems, Modifications, and Adjuvant-Antigen Codelivery. Vaccines.

[48] Polhemus M.E., Remich S.A., Ogutu B.R., Waitumbi J.N., Otieno L., Apollo S., Cummings J.F., Kester K.E., Ockenhouse C.F., Stewart A. (2009). Evaluation of RTS, S/AS02A and RTS, S/AS01B in adults in a high malaria transmission area. PLoS ONE.

[49] Agnandji S.T., Asante K.P., Lyimo J., Vekemans J., Soulanoudjingar S.S., Owusu R., Shomari M., Leach A., Fernandes J., Dosoo D. (2010). Evaluation of the safety and immunogenicity of the RTS, S/AS01E malaria candidate vaccine when integrated in the expanded program of immunization. J. Infect. Dis..

[50] Bejon P., Lusingu J., Olotu A., Leach A., Lievens M., Vekemans J., Mshamu S., Lang T., Gould J., Dubois M.C. (2008). Efficacy of RTS, S/AS01E vaccine against malaria in children 5 to 17 months of age. N. Engl. J. Med..

[51] Lell B., Agnandji S., von Glasenapp I., Haertle S., Oyakhiromen S., Issifou S., Vekemans J., Leach A., Lievens M., Dubois M.C. (2009). A randomized trial assessing the safety and immunogenicity of AS01 and AS02 adjuvanted RTS, S malaria vaccine candidates in children in Gabon. PLoS ONE.

[52] Olotu A., Moris P., Mwacharo J., Vekemans J., Kimani D., Janssens M., Kai O., Jongert E., Lievens M., Leach A. (2011). Circumsporozoite-specific T cell responses in children vaccinated with RTS, S/AS01E and protection against P falciparum clinical malaria. PLoS ONE.

[53] Owusu-Agyei S., Ansong D., Asante K., Kwarteng Owusu S., Owusu R., Wireko Brobby N.A., Dosoo D., Osei Akoto A., Osei-Kwakye K., Adjei E.A. (2009). Randomized controlled trial of RTS, S/AS02D and RTS, S/AS01E malaria candidate vaccines given according to different schedules in Ghanaian children. PLoS ONE.

[54] RTS, S (2015). Efficacy and safety of RTS, S/AS01 malaria vaccine with or without a booster dose in infants and children in Africa: Final results of a phase 3, individually randomised, controlled trial. Lancet.

[55] RTS, S (2014). Clinical Trials Partnership. Efficacy and safety of the RTS, S/AS01 malaria vaccine during 18 months after vaccination: A phase 3 randomized, controlled trial in children and young infants at 11 African sites. PLoS Med..

[56] Laurens M.B. (2020). RTS, S/AS01 vaccine (Mosquirix™): An overview. Hum. Vaccines Immunother..

[57] Olotu A., Fegan G., Wambua J., Nyangweso G., Leach A., Lievens M., Kaslow D.C., Njuguna P., Marsh K., Bejon P. (2016). Seven-Year Efficacy of RTS, S/AS01 Malaria Vaccine among Young African Children. N. Engl. J. Med..

[58] Penny M.A., Verity R., Bever C.A., Sauboin C., Galactionova K., Flasche S., White M.T., Wenger E.A., Van de Velde N., Pemberton-Ross P. (2016). Public health impact and cost-effectiveness of the RTS, S/AS01 malaria vaccine: A systematic comparison of predictions from four mathematical models. Lancet.

[59] Wagnew Y., Hagos T., Weldegerima B., Debie A. (2021). Willingness to Pay for Childhood Malaria Vaccine Among Caregivers of Under-Five Children in Northwest Ethiopia. Clin. Outcomes Res. CEOR.

[60] Aide P., Dobaño C., Sacarlal J., Aponte J.J., Mandomando I., Guinovart C., Bassat Q., Renom M., Puyol L., Macete E. (2011). Four year immunogenicity of the RTS, S/AS02(A) malaria vaccine in Mozambican children during a phase IIb trial. Vaccine.

[61] Campo J.J., Dobaño C., Sacarlal J., Guinovart C., Mayor A., Angov E., Dutta S., Chitnis C., Macete E., Aponte J.J. (2011). Impact of the RTS, S malaria vaccine candidate on naturally acquired antibody responses to multiple asexual blood stage antigens. PLoS ONE.

[62] Dobaño C., Campo J.J., Dobaño C., Sacarlal J., Guinovart C., Mayor A., Angov E., Dutta S., Chitnis C., Macete E. (2019). Differential Patterns of IgG Subclass Responses to Plasmodium falciparum Antigens in Relation to Malaria Protection and RTS, S Vaccination. Front. Immunol..

[63] Moncunill G., Mpina M., Nhabomba A.J., Aguilar R., Ayestaran A., Sanz H., Campo J.J., Jairoce C., Barrios D., Dong Y. (2017). Distinct Helper T Cell Type 1 and 2 Responses Associated With Malaria Protection and Risk in RTS, S/AS01E Vaccinees. Clin. Infect. Dis..

[64] Moorthy V.S., Ballou W.R. (2009). Immunological mechanisms underlying protection mediated by RTS, S: A review of the available data. Malar. J..

[65] White M.T., Verity R., Griffin J.T., Asante K.P., Owusu-Agyei S., Greenwood B., Drakeley C., Gesase S., Lusingu J., Ansong D. (2015). Immunogenicity of the RTS, S/AS01 malaria vaccine and implications for duration of vaccine efficacy: Secondary analysis of data from a phase 3 randomised controlled trial. Lancet Infect. Dis..

[66] Kazmin D., Nakaya H.I., Lee E.K., Johnson M.J., van der Most R., van den Berg R.A., Ballou W.R., Jongert E., Wille-Reece U., Ockenhouse C. (2017). Systems analysis of protective immune responses to RTS, S malaria vaccination in humans. Proc. Natl. Acad. Sci. USA.

[67] Laurens M.B. (2018). The Promise of a Malaria Vaccine-Are We Closer?. Annu. Rev. Microbiol..

[68] Ishizuka A.S., Lyke K.E., DeZure A., Berry A.A., Richie T.L., Mendoza F.H., Enama M.E., Gordon I.J., Chang L.J., Sarwar U.N. (2016). Protection against malaria at 1 year and immune correlates following PfSPZ vaccination. Nat. Med..

[69] Seder R.A., Chang L.J., Enama M.E., Zephir K.L., Sarwar U.N., Gordon I.J., Holman L.A., James E.R., Billingsley P.F., Gunasekera A. (2013). Protection against malaria by intravenous immunization with a nonreplicating sporozoite vaccine. Science.

[70] Lyke K.E., Ishizuka A.S., Berry A.A., Chakravarty S., DeZure A., Enama M.E., James E.R., Billingsley P.F., Gunasekera A., Manoj A. (2017). Attenuated PfSPZ Vaccine induces strain-transcending T cells and durable protection against heterologous controlled human malaria infection. Proc. Natl. Acad. Sci. USA.

[71] Sissoko M.S., Healy S.A., Katile A., Omaswa F., Zaidi I., Gabriel E.E., Kamate B., Samake Y., Guindo M.A., Dolo A. (2017). Safety and efficacy of PfSPZ Vaccine against Plasmodium falciparum via direct venous inoculation in healthy malaria-exposed adults in Mali: A randomised, double-blind phase 1 trial. Lancet Infect. Dis..

[72] Epstein J.E., Tewari K., Lyke K.E., Sim B.K., Billingsley P.F., Laurens M.B., Gunasekera A., Chakravarty S., James E.R., Sedegah M. (2011). Live attenuated malaria vaccine designed to protect through hepatic CD8⁺ T cell immunity. Science.

[73] Collins K.A., Snaith R., Cottingham M.G., Gilbert S.C., Hill A. (2017). Enhancing protective immunity to malaria with a highly immunogenic virus-like particle vaccine. Sci. Rep..

[74] Regules J.A., Cicatelli S.B., Bennett J.W., Paolino K.M., Twomey P.S., Moon J.E., Kathcart A.K., Hauns K.D., Komisar J.L., Qabar A.N. (2016). Fractional Third and Fourth Dose of RTS, S/AS01 Malaria Candidate Vaccine: A Phase 2a Controlled Human Malaria Parasite Infection and Immunogenicity Study. J. Infect. Dis..

[75] Ledford H. (2021). Malaria vaccine wows and seeds COVID-19 vaccine effort. Nat. Biotechnol..

[76] Islam S.U., Shehzad A., Ahmed M.B., Lee Y. (2020). Intranasal Delivery of Nanoformulations: A Potential Way of Treatment for Neurological Disorders. Molecules.

[77] Mallory K.L., Taylor J.A., Zou X., Waghela I.N., Schneider C.G., Sibilo M.Q., Punde N.M., Perazzo L.C., Savransky T., Sedegah M. (2021). Messenger RNA expressing PfCSP induces functional, protective immune responses against malaria in mice. Npj Vaccines.

[78] Al-Suhaimi E.A., Aljafary M.A., Alkhulaifi F.M., Aldossary H.A., Alshammari T., Al-Qaaneh A., Aldahhan R., Alkhalifah Z., Gaymalov Z.Z., Shehzad A. (2021). Thymus Gland: A Double Edge Sword for Coronaviruses. Vaccines.

[79] Wahid F., Shehzad A., Khan T., Kim Y.Y. (2010). MicroRNAs: Synthesis, mechanism, function, and recent clinical trials. Biochim. Biophys. Acta.

